# Co-culture of hWJMSCs and pACs in double biomimetic ACECM oriented scaffold enhances mechanical properties and accelerates articular cartilage regeneration in a caprine model

**DOI:** 10.1186/s13287-020-01670-2

**Published:** 2020-05-19

**Authors:** Yu Zhang, Chunxiang Hao, Weimin Guo, Xiaoyu Peng, Mingjie Wang, Zhen Yang, Xu Li, Xueliang Zhang, Mingxue Chen, Xiang Sui, Jiang Peng, Shibi Lu, Shuyun Liu, Quanyi Guo, Qing Jiang

**Affiliations:** 1 Institute of Orthopaedics, Chinese PLA General Hospital, 28 Fuxing Road, Haidian District, Beijing, 100853 China; 2 Beijing Key Lab of Regenerative Medicine in Orthopaedics, 28 Fuxing Road, Haidian District, Beijing, 100853 China; 3 Key Laboratory of Musculoskeletal Trauma & War Injuries, PLA, 28 Fuxing Road, Haidian District, Beijing, 100853 China; 4 Department of Sports Medicine and Adult Reconstructive Surgery, Nanjing Drum Tower Hospital, The Affiliated Hospital of Nanjing University Medical School, 321 Zhongshan Road, Gulou District, Nanjing, 210008 China; 5 Institute of Anesthesia, Chinese PLA General Hospital, 28 Fuxing Road, Haidian District, Beijing, 100853 China; 6 Department of Geriatrics, Nanjing Drum Tower Hospital, The Affiliated Hospital of Nanjing University Medical School, 321 Zhongshan Road, Gulou District, Nanjing, 210008 China; 7 School of Medicine, Naikai University, Tianjin, 300071 China; 8 Shanxi Traditional Chinese, No. 46 Binzhou west Street, YingZe District, Taiyuan, 030001 China

**Keywords:** Acellular cartilage extracellular matrix-oriented scaffold, Human umbilical cord Wharton’s jelly-derived mesenchymal stem cell, Primary cartilage cell, Co-culture system

## Abstract

**Background:**

The dedifferentiation of chondrocytes and the unstable chondrogenic differentiation status of pluripotent mesenchymal stem cells (MSCs) are immense issues in cell-based articular cartilage repair and regenerative strategies. Here, to improve the cartilage characteristics of seed cells, a double biomimetic acellular cartilage extracellular matrix (ACECM)-oriented scaffold was used to mimic the cartilage microenvironment for human umbilical cord Wharton’s jelly-derived MSCs (hWJMSCs) and primary cartilage cells (pACs) to regenerate hyaline cartilage.

**Methods:**

A double biomimetic ACECM-oriented scaffold was created from the cartilage extracellular matrix of pig articular cartilage using pulverization decellularization freeze-drying procedures. hWJMSCs and pACs were co-cultured at ratios of 50:50 (co-culture group, ACCC), 0:100 (ACAC group) and 100:0 (ACWJ group) in the ACECM-oriented scaffold, and the co-culture system was implanted in a caprine model for 6 months or 9 months to repair full-thickness articular cartilage defects. The control groups, which had no cells, comprised the blank control (BC) group and the ACECM-oriented scaffold (AC) group. Gross morphology and magnetic resonance imaging (MRI) as well as histological and biomechanical evaluations were used to characterize the cartilage of the repair area.

**Results:**

Relative to the control groups, both the gross morphology and histological staining results demonstrated that the neotissue of the ACCC group was more similar to native cartilage and better integrated with the surrounding tissue. Measurements of glycosaminoglycan content and Young’s modulus showed that the repair areas had more abundant cartilage-specific content and significantly higher mechanical strength in the ACCC group than in the control groups, especially at 9 months. On MRI, the T2-weighted signal of the repair area was homogeneous, and the oedema signal disappeared almost completely in the ACCC group at 9 months. HLA-ABC immunofluorescence staining demonstrated that hWJMSCs participated in the repair and regeneration of articular cartilage and escaped surveillance and clearance by the caprine immune system.

**Conclusion:**

The structure and components of double biomimetic ACECM-oriented scaffolds provided a cartilage-like microenvironment for co-cultured seed cells and enhanced the biomechanics and compositions of neotissue. This co-culture system has the potential to overcome the dedifferentiation of passage chondrocytes and the unstable chondrogenic differentiation status of MSCs.

## Background

Articular cartilage injury, usually caused by engaging in exercise, professional training and games, trauma due to accident, or being overweight, has become increasingly common among young populations and professional athletes. A retrospective study of 5233 knee arthroscopies by Lukasik et al. reported that over 57% of patients were diagnosed with a cartilage lesion [1]. In addition, the overall prevalence of articular cartilage injury in athletes (36%) is more than twice the rate in the general population [2]. However, cartilage rarely self-heals because it lacks vascular and lymph tissue to provide nutrients [3]; thus, osteoarthritis generally develops within 5–20 years [4, 5]. Cell-based treatment strategies are promising methods to repair and regenerate articular cartilage and to stop the progression of osteoarthritis [6, 7].

However, the histological characteristics of neotissue are usually highly inferior to those of native articular cartilage mainly because of the unstable chondrogenic differentiation status of mesenchymal stem cells (MSCs), which results in the presence of hypertrophic or calcified chondrocytes [8–11]. In addition, for autologous chondrocyte implantation (ACI) or matrix -induced chondrocyte implantation (MACI), chondrocytes also face some problems, including limited cartilage source to isolate primary articular cartilage cells (pACs) [8, 12] and dedifferentiation after several passages [13]. In recent decades, it has been emphasized that the environment plays a critical role in the regulation of cell behaviour [14–16]. Co-culture has been explored to promote the chondrogenic induction of MSCs and to maintain the native phenotype of chondrocytes by bio-stimulation via cell-cell interactions in co-culture systems [14, 17, 18]. In addition, co-culture reduces the required amount of cartilage tissue, decreases the in vitro culture time and lowers the degree of chondrocyte degeneration [13]. However, some studies showed that co-culture merely promoted a unidirectional trophic effect of MSCs on chondrocytes [19, 20]. These differences are probably attributable to the complex microenvironment, which (i) comprises a milieu of biochemical, biomechanical and bioelectrical signals derived from surrounding cells, the extracellular matrix (ECM) and soluble factors and (ii) regulates cell metabolism and function.

In vitro, a co-culture system was constructed using unpassaged pACs possessing their native phenotype and an ACECM-oriented scaffold retaining native cartilage tissue components and oriented structures [21–23]; hWJMSCs were displayed as an alternative cells source for cartilage tissue engineering because of distinct chondrogenic potential, highly proliferative activity and safety for patients [24, 25]. In vitro, hWJMSCs were co-cultured with chondrocytes and successfully differentiated into chondrocytes, as shown by the significant upregulation of collagen II and aggrecan genes [26]. In gross morphology, engineered tissue was translucent and resilient, similar to hyaline cartilage tissue. Despite these findings, the regeneration and repair conditions after the implantation of engineered tissue into cartilage defects in vivo remain unknown. Here, we evaluated whether the co-culture of hWJMSCs and pACs in a double biomimetic ACECM-oriented scaffold could achieve a joint surface that mimics the native state as closely as possible and return the articular cartilage to its natural biomechanics and compositions.

## Materials and methods

### Preparation of seed cells and construction of tissue-engineered cartilage in vitro

The collection of three healthy human umbilical cords for this study was approved by the Ethics Committee of the General Hospital of the People’s Liberation Army (PLA), and written informed consent was provided by all mothers. The detailed protocols for the isolation and culture of hWJMSCs and pACs have been previously described [26, 27]. According to previously described protocols, hWJMSCs were isolated using micromass mesenchymal tissue adhesion on a culture dish containing Dulbecco’s modified Eagle’s medium/-Ham’s F 12 (Sigma, USA) nutrient medium (DMEM/F12) (containing 10% foetal bovine serum (FBS) (Corning, USA), 100 mg/ml penicillin, 10 mg/ml streptomycin and 250 mg/ml amphotericin B). At passage 3, a subset of the isolates was prepared for co-culture with pACs. pACs were isolated from slices of goat shoulder articular cartilage by cutting them into microscopic-pieces and digesting them using 0.15% type II collagenase for 2 h at 37 °C. Then, the individual cells and cell sheet were collected by centrifugation at 250×*g* for 5 min at room temperature. The cells were resuspended in DMEM/F12 culture medium supplemented with 10% FBS and antibiotics (100 mg/ml penicillin, 10 mg/ml streptomycin and 250 mg/ml amphotericin B) and cultured in 25-cm^2^ culture flasks with 5% CO_2_ in a 37 °C incubator. The pACs were collected for construction of the co-culture system at approximately 80% confluence.

A three-dimensional (3D)-oriented scaffold was made from acellular ECM using the freeze-drying technique previously described [21]. After cross-linking by dehydrothermal and water-soluble carbodiimide treatment, oriented scaffolds with a diameter of 6 mm and a thickness of 1 mm were produced and sterilized by ^60^Co-γ irradiation (at 5 mrad). A total of 10^7^ pACs and hWJMSCs were mixed in three different ratios: 100:0, 0:100 and 50:50 in 100 μl DMEM/F12 medium. Then, the cell suspension was seeded into a 3D-oriented scaffold and incubated for 2 h at 37 °C with 5% CO_2_ to promote cell adhesion to the scaffolds. Next, cell-scaffold complexes were transferred to 6-well cell culture plates, and 5 ml of culture medium (DMEM/F12, 10% FBS, 100 mg/ml penicillin, 10 mg/ml streptomycin and 250 mg/ml amphotericin B) was added. Tissue-engineered cartilage was acquired after cultivation for 3 weeks at 37 °C under 5% CO_2._ The gross morphology was evaluated according to size, colour and shape.

### Cell viability and distribution assessment in the 3D-oriented scaffolds

Dead/live staining was used to analyse cell viability after culturing in the scaffolds for 3 days. Briefly, cell-scaffold complexes were stained with FDA (fluorescein diacetate, 5 μg/ml, Sigma) and PI (propidium iodide, 5 μg/ml, Sigma) for 5 min and rinsed 3 times with PBS (phosphate-buffered solution, pH 7.4). The samples were immediately visualized using a laser scanning confocal microscope (Olympus FV 1200, Japan) [26]. Red and green fluorescence indicate dead and live cells, respectively.

To observe the distribution of seed cells in the 3D-oriented scaffold, scanning electron microscopy (SEM) was applied to view the samples in the sagittal plane and in cross sections after culturing for 3 weeks. All samples were fixed in 2.5% glutaraldehyde, dehydrated in a series of graded ethanol to 100% ethanol, treated with hexamethyldisilazane and sputter-coated with gold-palladium before observation [21].

### In vivo experiments using the engineered tissue

To verify the effectiveness and safety of engineered tissue, the ACCC (pACs: hWJMSCs, 50:50) group was used to repair full-thickness articular cartilage defects of the femoral condyles in a caprine model. The animal experimental procedures followed the standards for the care and use of laboratory animals and were approved by the Institutional Animal Care and Use Committee of the Chinese PLA General Hospital. Three healthy goats were randomly assigned to each of the following groups per time point: a blank control group (BC, full-thickness articular cartilage defects without any treatment), an ACECM-oriented scaffold group (AC, treatment with ACECM-oriented scaffolds), a pACs+ACECM-oriented scaffold group (ACAC, treatment with pACs and ACECM-oriented scaffolds), a hWJMSCs+ACECM-oriented scaffold group (ACWJ, treatment with hWJMSCs and ACECM-oriented scaffolds) and a ACCC group (ACCC, treatment with hWJMSCs, pACs and ACECM-oriented scaffolds). Goats (white, male, 30 ± 5 kg,) were treated with sumianxin II (0.1 mL/kg) and ketamine (4 mg/kg) by intramuscular injection. After location in the supine position on operating table, sterilizing knee joint and spreading the drapes, the medical parapatellar longitudinal skin incision is 8–10-cm long. The capsule of the knee is exposed, and longitudinal-shaped capsulotomy is performed. Lateral dislocation of the patella and knee flexion at 120° was performed, and then the medical and lateral femoral condyles are exposed. Full-thickness articular cartilage defects in femoral condyles were made using a 6.5-mm-diameter corneal trephine under sterile conditions, while preserving the subchondral bone. Penicillin sodium was administered for 3 days to prevent infection. The goats were allowed to move freely in a comfortable environment after surgery.

### 7.0 T high-resolution magnetic resonance imaging evaluation

At 6 or 9 months after surgery, all goats were euthanized by intravenous pentobarbital overdose. MRI images of the entire fresh knee joints were obtained immediately using a volume coil on a laboratory 7.0 T MRI scanner (Bruker Biospec, Germany). T2-weighted spin-echo images with fat suppression were obtained in the sagittal, frontal and transverse planes. The imaging parameters were as follows: slice thickness (1.0 mm), echo time (36 ms), repetition time (5109 ms), flip angle (90°), acquisition time (18 min) and field of view (75 × 75 mm). All images were scored independently according to the International Cartilage Repair Society (ICRS) Whole-Organ MRI Score (WORMS) of the knee in OA by an experienced radiologist specialized in musculoskeletal medicine and blinded to the group assignments [28].

### Gross morphology and scoring

After MRI, the whole knee joints, including the femoral condyles, the corresponding meniscus and tibia plateau, and the surrounding synovial membrane, were exposed, photographed and grossly evaluated. The repair tissues were examined macroscopically and scored by three blinded observers using the ICRS scoring system for cartilage repair, including the degree of repair, integration, surface regularity and overall judgement [29].

### Histological assessment and scoring

Cartilage repair areas were fixed in 4% buffered formalin for 1 week, and decalcified in 10% EDTA for 4 weeks. After dehydration, repair tissues were embedded in paraffin and sectioned into 8-μm slices. Histology sections were stained with haematoxylin and eosin (H&E) and safranin “O” [27]. Three experienced pathologists scored the repair tissue in a blinded manner using the ICRS scoring system, which includes cartilage thickness, surface regularity, integration with adjacent host cartilage, cell morphology and matrix staining [30].

### Immunohistochemical staining

After deparaffinization, rehydration and antigen retrieval using boiled citric acid, tissue slices were incubated in primary antibodies for collagen type I (ab23446, Abcam, England) and collagen type II (ab34712, Abcam, England) at 4 °C overnight. Then, the primary antibody was washed away with PBS, and a MaxVision™ Detection System DAB kit (MaiXin Bio, China) was used to measure the colour according to the manufacturer’s protocol.

### Nanoindentation test and glycosaminoglycan analyses

To assess the biomechanical properties of the repair tissue, Young’s elastic modulus was measured using the In Situ Nanomechanical Test System (Hysitron, USA) in medium at room temperature. The radius of curvature of the cospherical diamond probe tip was 100 μm. The trapezoidal load function was used on each indent site with loading (10 s), holding (5 s) and unloading (10 s). The cylindrical loading device was positioned perpendicular to the repair area and advanced forward by 2000 nm at 200 nm/s.

The repair tissue was immediately extracted by the corneal trephine. The samples were crushed, and Grocott’s Methenamine Silver (GMS) Kit (Genmed Scientifics Inc., USA) was used to test the glycosaminoglycan (GAG) content according to manufacturer’s instructions. The GAG content (μg/sample) was calculated as a component of the histological analysis.

### HLA-ABC immunofluorescence staining

To determine whether hWJMSCs participated in the repair and regeneration of articular cartilage, a cell tracking technique was applied via HLA-ABC immunofluorescence staining. Briefly, the slices were dewaxed and dehydrated and the antigen was retrieved. Then, the slices were placed in 1/100 HLA-ABC primary antibody (DCABH-9508, Abcam, England) at 4 °C overnight. After washing away the primary antibody, the slices were incubated for 1 h with green fluorescent secondary antibody and for 5 min with Hoechst 33258 nuclear stain, and images were captured using an Olympus fluorescence microscope.

### Statistical analysis

Data were analysed using SPSS 18.0 (SPSS Inc., Chicago, IL, USA). The quantified values are reported as the means ± standard deviation. One-way analysis of variance (ANOVA) with the Student-Newman-Keuls (SNK) test was used to determine significant differences between the two groups, *n* = 3. A value of *P* < 0.05 was considered to indicate a significant difference.

## Results

### Gross morphology of the tissue-engineered cartilage, cell viability and distribution in the 3D-oriented scaffold

**Fig. 1:**
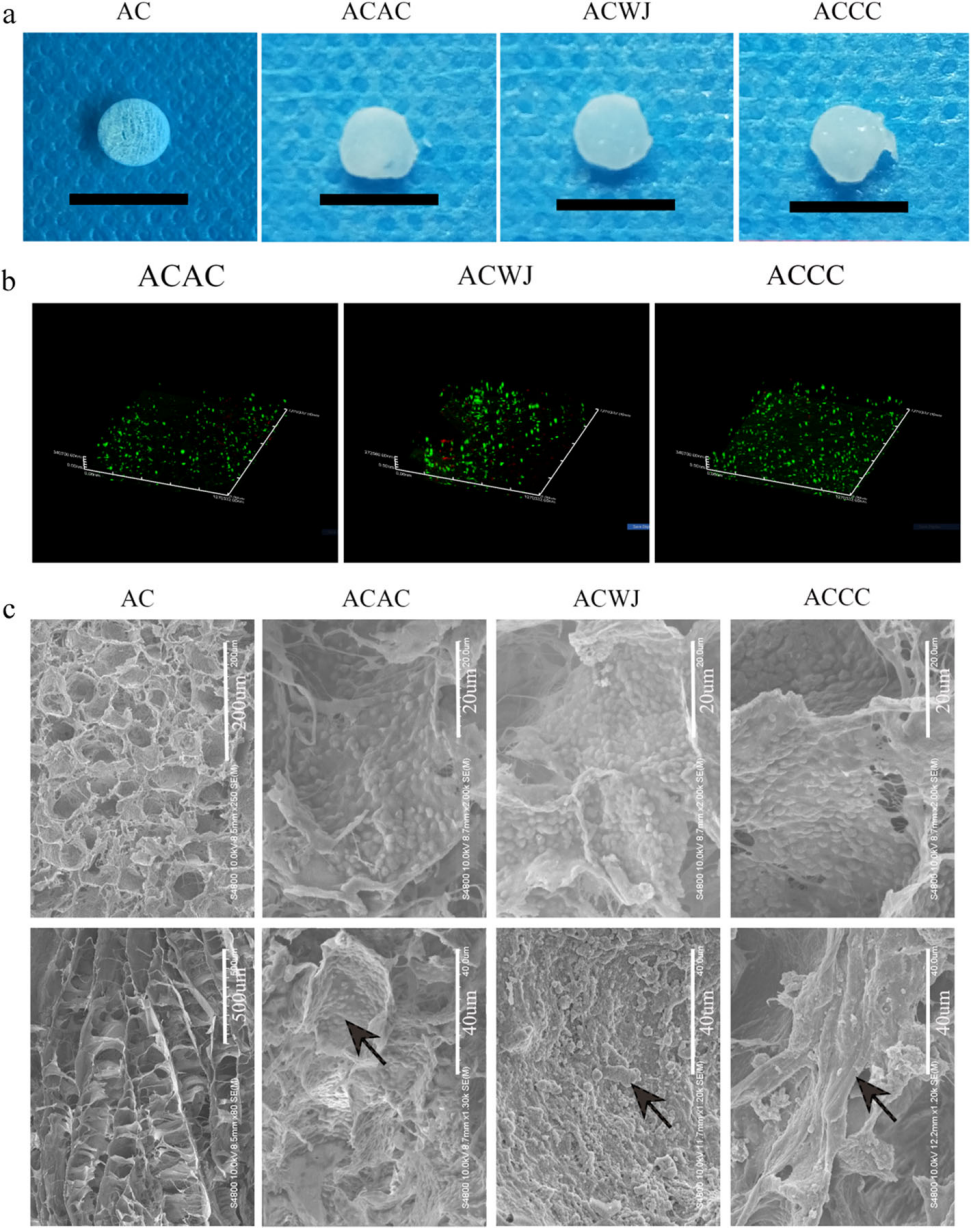
Hyaline cartilage-like gross morphology and directional distribution of cells and ECM after co-culture in ACECM-oriented scaffold. **a** After culture for 3 weeks, the 3D ACECM-oriented scaffolds were filled with cells and extracellular matrix in the ACAC, ACWJ and ACCC groups, and the appearance of the engineered tissue was semitransparent and glossy. The scale of black bar is 1 cm. **b** Dead/live staining under 3D confocal fluorescence microscopy. The 3D-oriented scaffolds are mainly filled with green fluorescent cells (living cells), with just a few red fluorescence cells (dead cells) present after cultivation for 3 days. **c** SEM results. The upper row shows the high-power (HP) field, and the lower row shows the low-power (LP) field; AC group, the upper and lower pictures are cross-sectional and sagittal plane views, respectively. In the ACAC, ACWJ and ACCC groups, the HP field shows cells distributed in holes, and the LP field shows the topological structure of the ECM; black arrows indicate the topological microstructure of ECM secreted by cells

After co-culture in ACECM-oriented scaffolds for 3 weeks in vitro, gross morphology characteristics of the tissue-engineered cartilage, including appearance, size and colour, were evaluated. In the cell-scaffold groups (including the ACAC, ACWJ and ACCC groups), the pores were almost filled with cells and ECM, and the size of the constructs was larger than that of the AC group. The constructs in the cell-scaffold groups appeared glossier and more semi-transparent than those in the AC group, and they had an appearance similar to native cartilage tissue (Fig. 1a).

After seeding into scaffolds for 3 days, cell viability was assessed using dead/live staining. Confocal microscopy showed that green fluorescent cells (living cells) were spread out in the 3D-oriented scaffold and were the main component in each group, even though some red fluorescent cells (dead cells) were dispersed in the scaffold. Thus, the 3D ACECM-oriented scaffold did not display significant cytotoxicity (Fig. 1b).

To explore the microstructure of the cell-scaffold, SEM was used to analyse the distribution of cells and the extracellular topological structure in the scaffold. The scaffold exhibited a honeycomb structure on cross-sections and stacked compartments on the sagittal plane. Among the cell-scaffold groups, the ACWJ group showed a high cell density, while the cells were sparse in the ACAC group. In view of the topological microstructure of ECM secreted by cells, the ACCC group presented thick and continuous connectivity among pores, while the ACWJ and ACAC groups had short and slim morphology, which is critical to guarantee strong mechanical properties, such as pressure and shear forces (Fig. 1c).

### In vivo experiments

One goat died during surgery because of an anaesthetic overdose, and another goat’s knee (in the ACWJ group) became infected on the 8th day after the operation but was later cured. Excluding the cases of infection and death, the remaining goats were included in the results analysis.

### Neotissue in the ACCC group with mature cartilage MRI signal and complete subchondral bone

**Fig. 2:**
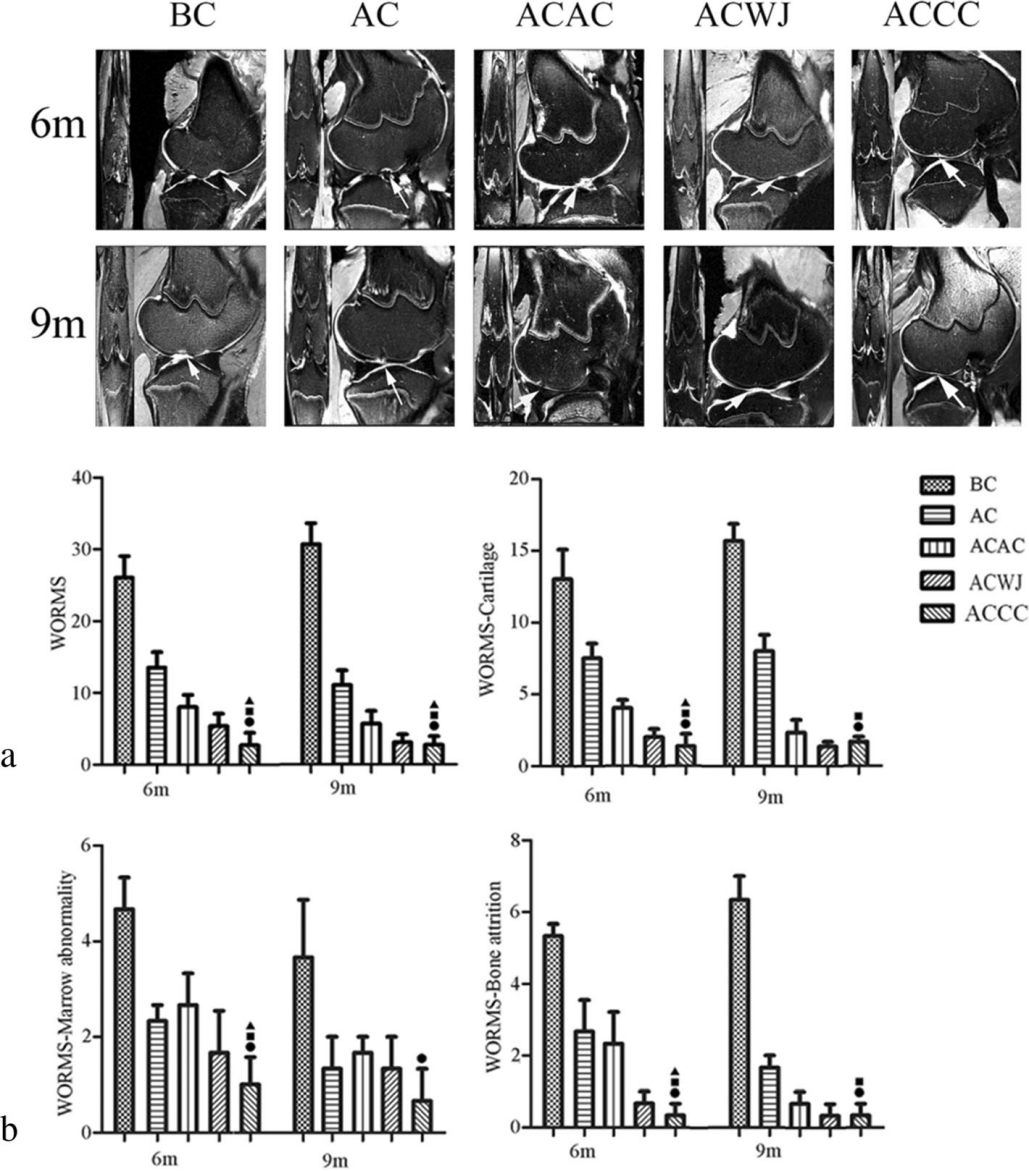
The ACCC group achieved early mature hyaline cartilage regeneration. **a** T2-weight spin-echo MRI with fat suppression in the sagittal plane and the coronal plane for each group at 6 months (6 m) and 9 months (9 m). Arrows indicate the defect region. **b** The scores were derived for five groups using the International Cartilage Repair Society (ICRS) Whole-Organ MRI Score (ICRS-WORMS) of the knee in OA. WORMS indicates the average score of the whole knee at 6 months (6 m) and 9 months (9 m). WORMS-MA, WORMS-Marrow abnormality: WORMS-BA, WORMS-bone attrition; WORMS-CA, WORMS-cartilage; *T* test was used for the data analysis and all data are expressed as the mean ± standard deviation (*n* = 6). Black circle, black square, black triangle and black star indicate significant differences between the ACCC group and the BC, AC, ACAC, and ACWJ groups, respectively. *P* < 0.05 indicates a statistically significant difference

MRI of the fresh whole knee joints of the ACCC group at 6 months showed that the damaged joints were almost completely filled with neotissue, and their surfaces were smooth. However, some cavities were still found in the repair area in the ACAC group and the ACWJ group, and obvious defects were still present in the AC group and the BC group. At 9 months, the neotissue was uniform and similar to that of native cartilage, and the oedema signal of subchondral bone had completely disappeared. The defects in the ACWJ group, the AC group and the ACAC group were filled with neotissue, but the MRI results showed that some joint effusion or immature cartilage signal existed (Fig. 2a).

Regarding the WORMS results of the knee, the cartilage signal and morphology scores were better in the ACWJ group (6 months, 2.0 ± 1.0; 9 months, 1.3 ± 0.6) and the ACCC group (6 months, 1.3 ± 1.5; 9 months, 1.6 ± 0.6) than in the AC group or the BC group. The ACAC group exhibited improved neotissue quality at 9 months compared with that at 6 months. With respect to subchondral bone attrition, the BC group and the AC group were markedly concave (5.3 ± 0.6; 6.3 ± 1.2) and slightly concave (2.8 ± 0.7; 1.7 ± 0.4) at 6 months and 9 months, respectively. Compared with the other groups, the marrow abnormality scores were lowest in the ACCC group, with < 25% abnormal regions. The total WORMS results revealed the following: the best neotissue quality and repair was found in the knees from the ACCC group and the ACWJ group, followed by the ACAC group and the AC group. The BC group had the worst WORMS results, with no any repair or regeneration observed (Fig. 2b).

### The ACCC group achieved faster repair and higher ICRS-MECR scores than the other groups

**Fig. 3:**
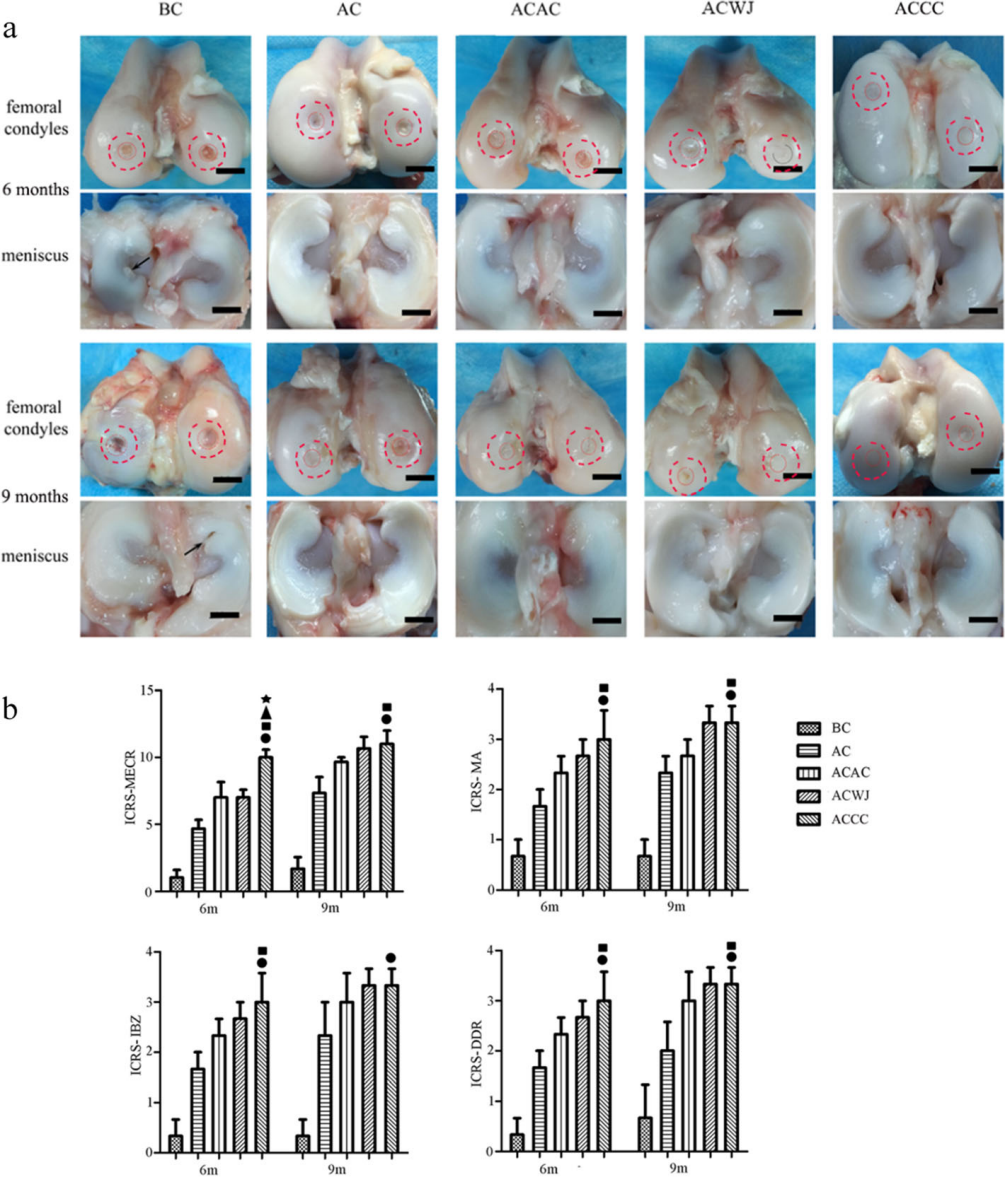
The repair area was smooth and full in the ACCC group. Gross morphology of the femoral condyle, the counterpart meniscus and the tibia platform and the results of International Cartilage Repair Society (ICRS) scoring system. **a** The red-dotted circles show the defects and the repair area; the black arrows and the black bars indicate the torn meniscus and a 1-cm scale, respectively. **b** ICRS scoring system for macroscopic evaluation of cartilage repair, ICRS-ORA, ICRS-overall repair assessment; ICRS-MA, ICRS-macroscopic appearance; ICRS-IBZ, ICRS-integration to border zone; ICRS-DDR, ICRS-degree of defect repair; *n* = 6, data are expressed as the mean ± standard deviation; black circles, black squares, black triangles and black stars indicate significant differences between the ACCC and the BC, AC, ACAC and ACWJ groups, respectively. *P* < 0.05 indicates a statistically significant difference

At 6 months, the cartilage defects were not completely repaired with neotissue in any group. However, the ACCC group was the only group in which a full-thickness cartilage defect was not observed. At 9 months, the repair area was plump and glossy because of the regeneration of neotissue in the ACCC group and the ACWJ group, but a local area of partial-thickness defects intermixed with areas of normal thickness still existed in the AC group and the ACAC group. The BC group showed almost no repair, or the depth of the defects was quite large (Fig. 3a).

The ICRS-MECR results showed that the repair and regeneration in the ACCC group (6 months, 10.0 ± 1.0; 9 months, 11.0 ± 1.7) was significantly better than that in the ACAC group (6 months, 7.0 ± 2.0; 9 months, 9.7 ± 0.6), the AC group (6 months, 4.7 ± 1.2; 9 months, 7.3 ± 2.1) and the BC group (6 months, 1.1 ± 0.4; 9 months, 1.5 ± 0.7), which highlights the intense integration with the border zone, the large degree of defect repair and the hyaline macroscopic appearance (Fig. 3b).

### Histological level revealed abundant hyaline cartilage components in the ACCC group

**Fig. 4:**
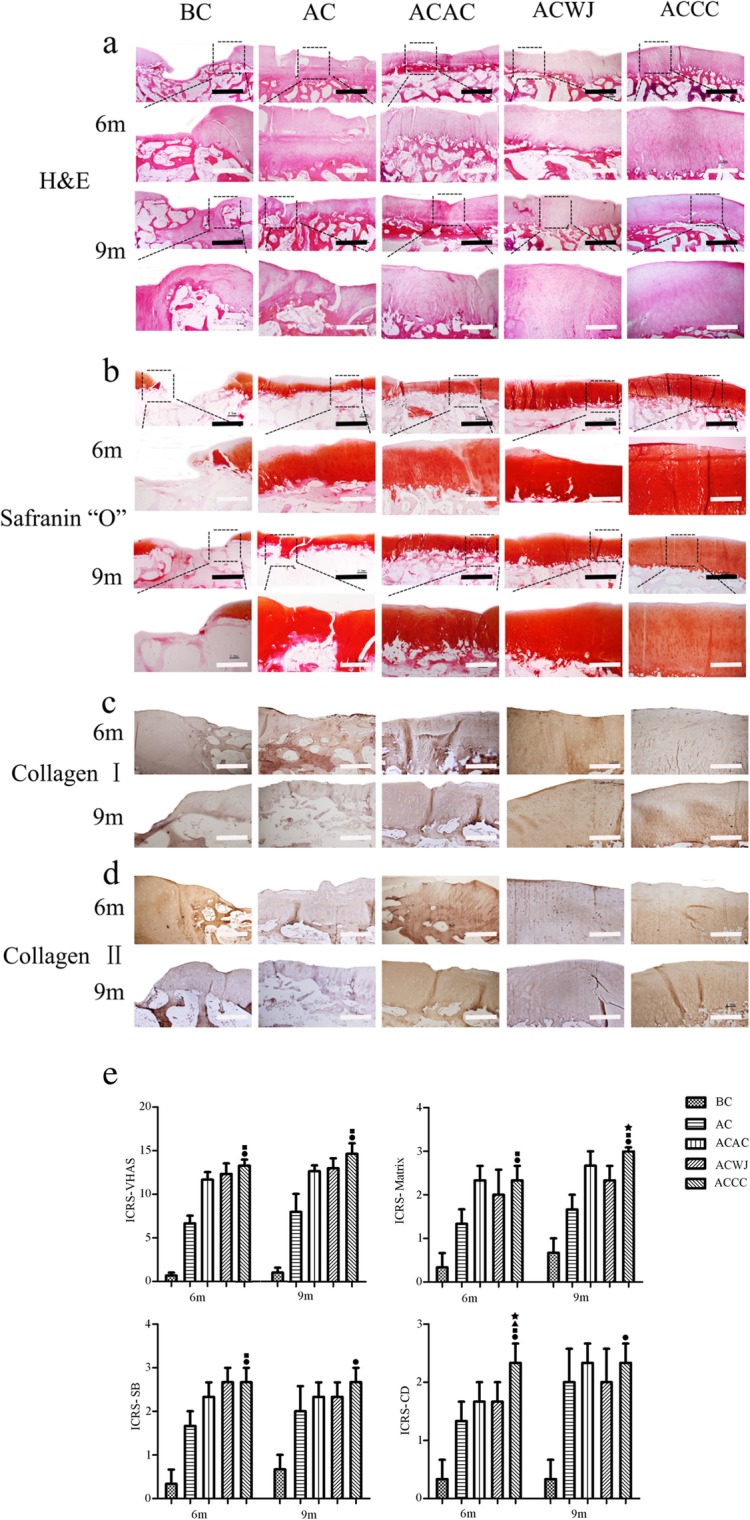
The neotissue characteristics in the ACWJ group were similar to the surrounding hyaline cartilage. **a**, **b** The results of H&E staining and Safranin “O” staining of the repair area of femoral condyles, respectively. The images below are the dotted box partial enlargement of the image above, the scales of black and white bar equal to 2 mm and 0.8 mm, respectively. **c**, **d** collagen I and collagen II immunohistochemistry staining of repair area of femoral condyles. **e** The results of the International Cartilage Repair Society Visual-Histological Assessment Scale for Cartilage Repair: ICRS-VHAS, ICRS- visual histological assessment scale; ICRS-SB, ICRS-subchondral bone; ICRS-CD, ICRS-cell distribution; ICRS-M, ICRS-matrix; *n* = 3. Data are expressed as the mean ± standard deviation; black circles, black squares, black triangles and black stars indicate the significant differences between the ACCC group and the BC, AC, ACAC and ACWJ groups. *P* < 0.05 indicates a statistically significant difference

The H&E results showed that at 6 months, neotissue padded the defects and covered and tightly aligned with the subchondral bone, and the thickness of the neotissue was similar to that of the surrounding cartilage in the ACCC group, whereas in the BC group, the subchondral bone was naked and concave. The neotissue in the AC group was thinner and less evenly distributed than in the cell-scaffold groups, including the ACAC group, the ACWJ group and the ACCC group. At 9 months, a distribution of mixed columnar clusters, a smooth surface and normal subchondral bone could be clearly observed in the ACCC group. The cartilage in the AC group did not integrate with the surrounding normal cartilage, and the cartilage in the ACAC group had an uneven surface (Fig. 4a).

Safranin “O” staining revealed that the ECM of neotissue had a sharp outline and showed different thicknesses and various grades of maturity. At 6 months, the ACWJ group and the AC group showed immature neotissue that appeared as loose fibrocartilage-like tissue. Compared with the ACCC group, other groups showed infrequent cartilage lacuna formation and dense matrix masses in the repair area at 9 months (Fig. 4b).

Immunohistochemical staining at 6 months showed that the ACCC group was negative for collagen I whereas the AC group and the ACWJ group were not. And collagen I at 9 months in the ACCC group was slightly higher than AC group. The ACCC group was significantly positive for collagen II at both 6 months and 9 months, while the control groups but the AC group showed negative staining results. Compared with the AC group, collagen II was less expressed in the ACCC group at 6 months, but there was no significant difference at 9 months (Fig. 4 c, d).

According to the ICRS-VHAS, the ACCC group (6 months, 13.3 ± 1.2; 9 months, 14.7 ± 2.1), the ACAC group (6 months, 11.7 ± 1.5; 9 months, 12.7 ± 1.2) and the ACWJ group (6 months, 11.7 ± 1.5; 9 months, 13.0 ± 2.0) achieved better tissue repair than the BC group (6 months, 0.7 ± 0.6; 9 months, 1.0 ± 1.0). The significant advantages of the ACCC group over the other cell-scaffold complex groups included the formation of mature hyaline cartilage-like ECM and a columnar or clustered cell distribution (Fig. 4e).

### Biomechanical and glycosaminoglycan quantitative analyses

**Fig. 5:**
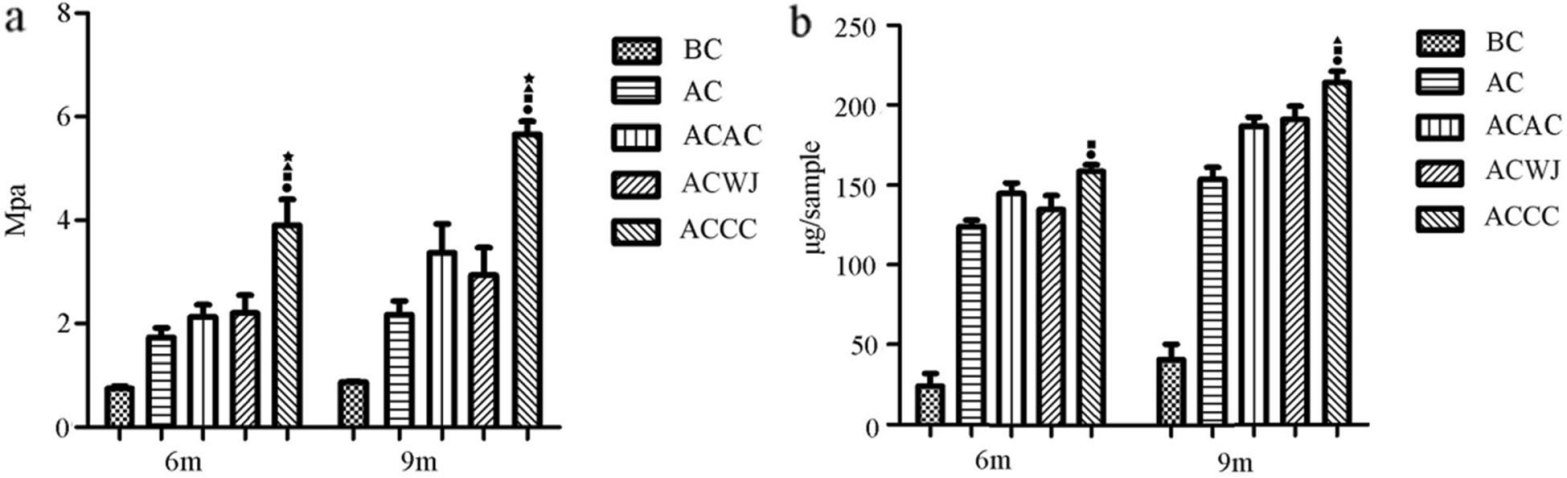
The biochemical and biomechanical characteristics of the ACCC group were significantly better than those of the other control groups. Quantitative analysis of articular cartilage repair. Biochemical and biomechanical evaluation was performed to quantify **a** Young’s modulus and **b** the glycosaminoglycan (GAG) content in the three samples. A *T* test was used for data analysis, and all data are expressed as the mean ± standard deviation, with *n* = 6. Black circles, black squares, black triangles and black stars indicate the significant differences between the ACCC group and the BC, AC, ACAC, and ACWJ groups. *P* < 0.05 indicates a statistically significant difference

As a critical functional indicator of articular cartilage, Young’s modulus was accurately measured using the nanoindentation technique. At 6 months, the Young’s modulus of the ACCC group (3.9 ± 0.9) was significantly higher than those of the other groups, including the ACWJ group (2.2 ± 0.6), the ACAC group (2.1 ± 0.4) and the AC group (1.7 ± 0.3). The difference between the ACCC group and cell-scaffold groups increased at 9 months (Fig. 5a).

The total GAG content per sample showed no significant difference among the cell-scaffold groups, but it was more abundant in the ACCC group at 6 months. The GAG content of the ACCC group was higher than that of the AC group at 9 months, while no significant difference was found between the ACAC group and the ACWJ group (Fig. 5b).

### hWJMSCs participated in the repair and regeneration of cartilage defects

**Fig. 6:**
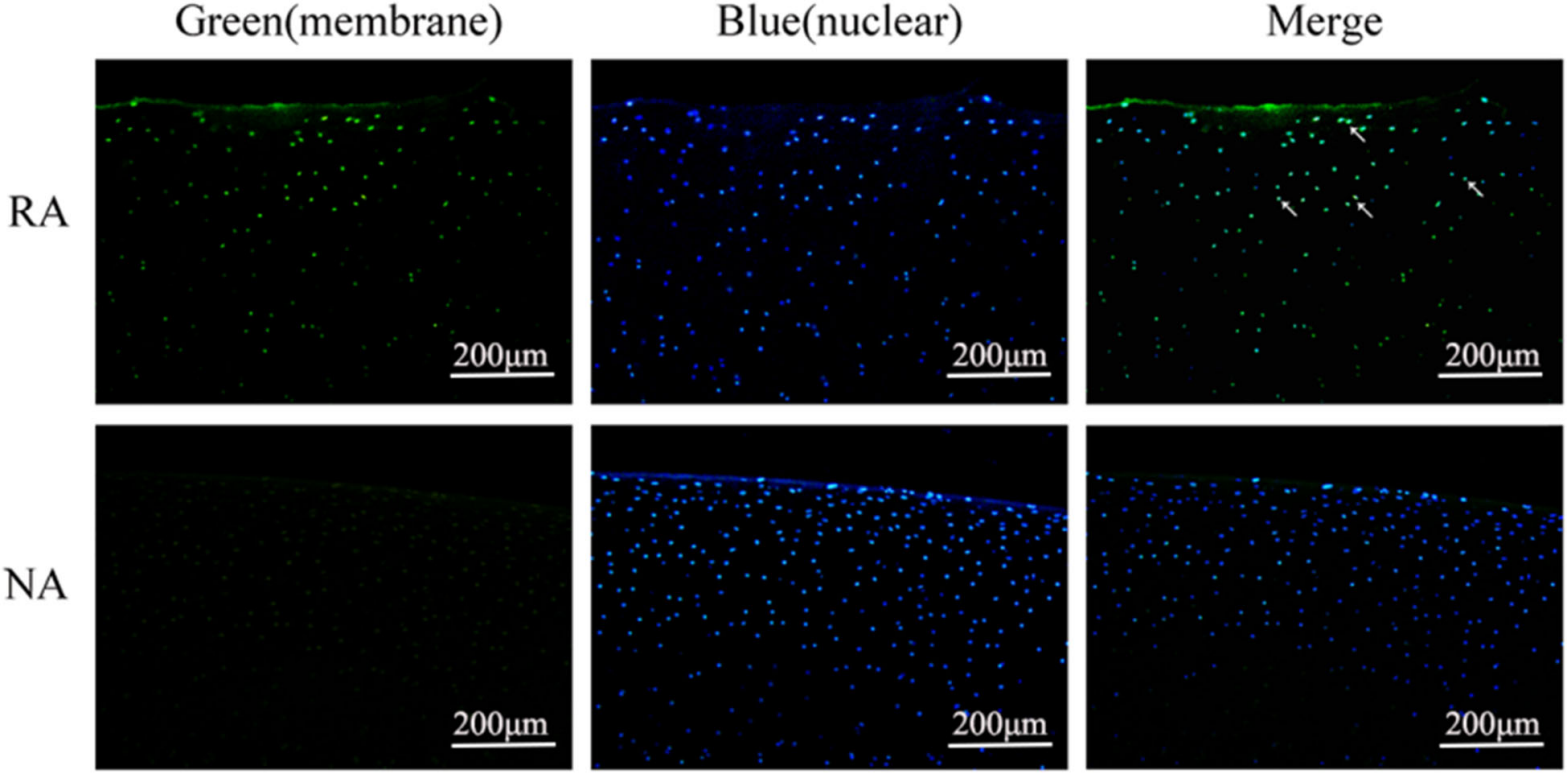
hWJMSCs participated in the repair and regeneration of articular cartilage. The results of immunofluorescence (IF) staining for HLA-ABC were used to track hWJMSCs in the ACCC group at 9 months. Green indicates positive immunofluorescence staining for HLA-ABC, and blue shows nuclear staining by Hoechest33258. The repair area (RA) shows positive staining results, while the normal area (NR) was negative

As a human leukocyte antigen I molecular marker, HLA-ABC immunofluorescence staining can provide evidence demonstrating that hWJMSCs are one part of the neotissue and evade from surveillance of the immune system. Green fluorescence can be observed in the repair area of the ACCC group, while it is absent in the normal area, indicating that hWJMSCs were present in the repair area and were an important component of neotissue (Fig. 6).

## Discussion

### A co-culture system provides a potential strategy for the repair and regeneration of cartilage

Articular cartilage injury is a common sports-related disease that severely affects subsequent achievements and career planning. A co-culture system provides a win-win strategy to eliminate issues related to chondrocytes and MSCs and facilitate high-quality cartilage regeneration. The results obtained in vivo indicated the following: MRI showed that there was no significant joint effusion or bone marrow oedema signal in the ACCC group, and the neotissue of the ACCC group showed a smooth surface and uniform signal. Gross morphology showed that the ACCC group successfully achieved complete repair and regeneration at 6 months, and the histological properties of the neotissue were highly similar to those of native articular cartilage, including the thickness of the neotissue, the columnar cell distribution, the formation of cartilage lacuna and the high expression of collagen II. Although the damaged cavities observed in the ACWJ group were also completely covered with neotissue at 6 months, both the Young’s modulus and GAG content per sample in the ACCC group were improved at 6 months and especially at 9 months, highlighting the superiority of the regeneration in this group. From these animal experiments, we conclude that the group implanted with only the ACECM-oriented scaffold, without any seed cells, required much more time for neotissue generation to occur. The HLA-ABC measurements also provided important data that indirectly confirmed that the hWJMSCs possessed low immunogenicity and escaped destruction by the immune system. This result is congruent with previous findings showing that hWJMSCs elicit a very low immune response [31–33].

### The biological microenvironment affects cellular behaviour and represents a potential repair mechanism elicited by engineered cartilage tissue

In this paper, engineered cartilage tissue was successfully constructed by culturing hWJMSCs in a simulated cartilage microenvironment (consisting of pACs and ACECM-oriented scaffolds), and this construct facilitated the repair and regeneration of full-thickness defects of the femoral condyle in a caprine model. The potential mechanisms may involve (i) the primitive hyaline cartilage phenotype and the improved activity of pACs or (ii) the stable chondrogenic differentiation of hWJMSCs because of mild and durable regulation of the biomimetic cartilage microenvironment, not the temporary and unique action of a chondrogenic inducer. The in vitro results from our previous study revealed that hWJMSCs in a co-culture system were successfully induced into chondrocytes without significant upregulation of collagen X [26]. Other researchers have also reported that hWJMSCs can inhibit hypertrophy and improve chondrogenesis, as articular chondrocytes secrete parathyroid hormone-related protein (PTHrP) in co-culture systems [11, 34]. In addition, hWJMSCs possess better chondrogenic differentiation capacity than bone marrow mesenchymal stem cells (BMSCs) [24, 35, 36]. These factors may contribute greatly to chondrogenic differentiation and the regeneration of articular cartilage via hWJMSCs. It was also demonstrated by other studies in vivo that co-culture of MSCs and chondrocytes possessed obviously potential to repair and regeneration of articular cartilage compared with monoculture [37, 38]. MSCs differentiated into chondrocytes under co-culture system microenvironment adding no inducers in these two studies, which is similar to our study.

In the co-culture system, hWJMSCs rather than pACs eliminate the obstacle of limited cartilage resources and prevent the dedifferentiation of chondrocytes after expansion in vitro [39–41]. Additionally, the oriented microstructure of the scaffold can direct collagen fibre and ECM-oriented arrangement, which also plays an important role in enhancing the biomechanical strength of engineered cartilage tissue [42, 43]; however, biomechanical testing was not performed due to the short cultivation time in vitro.

### Limitations

Some questions remain unanswered in this study, and further research needs to be carried out to elucidate the functional mechanisms in the co-culture system. For example, the specific cytokines secreted by pACs or hWJMSCs and their signalling pathways remain unclear; how they regulate the cellular biobehaviour is also unknown. It remains unknown what constitutes the optimal construct, including optimal cell number MSC to AC ratio and direct or indirect co-culture models. The results showed that in as long as 9 months, the longer term stability of this construct is unknown. Moreover, in this study on a large animal model, immunological rejection of hWJMSCs through the search of specific antibodies has not been investigated. The risk of spreading disease is another disadvantage for patients if hWJMSCs are derived from an allogeneic umbilical cord. These findings are very important for guiding the development and optimization of co-culture models and for constructing tissue-engineered cartilage in one step without chondrocytes and the clinical application of hWJMSCs in the future. As for the experimental model, two defects were made in medical and lateral femoral condyles of one knee joint, which probably influence each other’s repair results.

## Conclusion

Co-culture of hWJMSCs and pACs in a spatial structure and component biomimetic ACECM-oriented scaffold showed clear advantages: pACs maintained a good primitive chondrocyte phenotype, and hWJMSCs remained in a stable chondrogenic differentiation status in the biomimetic cartilage microenvironment, which greatly contributed to achieve high-quality regeneration of injured cartilage and yielded a product that was similar to the native articular cartilage in terms of its biomechanics and tissue components. Future studies should investigate the regulation mechanisms of underlying the cellular behaviour in this co-culture system and explore the efficacy and safety of this system for future clinical applications.

## Data Availability

The datasets used and/or analysed during the current study are available from the corresponding author on reasonable request.

## Abbreviations

ACECM: Acellular cartilage extracellular matrix
BMSCs: Bone marrow mesenchymal stem cells
DMEM/F12: Dulbecco’s modified Eagle’s medium/-Ham’s F 12
FBS: Foetal bovine serum
FDA: Fluorescein diacetate
GAG: Glycosaminoglycan
hWJMSCs: Human umbilical cord Wharton’s jelly-derived MSCs
ICRS: International Cartilage Repair Society
ICRS-VHAS: International Cartilage Repair Society Visual-Histological Assessment Scale
MSCs: Mesenchymal stem cells
OCT: Optimum cutting temperature
pACs: Primary articular cartilage cells
PBS: Phosphate-buffered solution
PI: Propidium iodide
PTHrP: Parathyroid hormone-related protein
WORMS: Whole-Organ MRI Score
